# Supplementary material for: Recyclable Thermoplastic Elastomer from Furan Functionalized Hairy Nanoparticles with Polystyrene Core and Polydimethylsiloxane Hairs
Source: Polymers (Basel). 2024 Nov 7;16(22):3117. doi: 10.3390/polym16223117 (PMC11598078; doi:10.3390/polym16223117)
Supplement: Supplementary file 1 [file polymers-16-03117-s001.zip › polymers-3255307-supplementary.pdf]

Supporting Document

Recyclable Thermoplastic Elastomer from Furan Functionalized Hairy Nanoparticles with Poly(styrene) Core and Poly(dimethylsiloxane) Hairs

Md Hanif Uddin[1], Sultan Alshali[1], Esam Alqurashi[1], Saber Alyoubi[1], Natalia Walters[1], Ishrat M. Khan[1]*
Department of Chemistry, Clark Atlanta University; Atlanta, Georgia 30314
***Correspondence: ikhan@cau.edu (I.K.)**

Synthesis of 2-vinylfuran:
Furfural (0.05 mole), anhydrous potassium carbonate (0.20 mole), methyl triphenylphosphonium bromide (0.05 mole), nitrobenzene (60 ml), and water (1 ml) were sequentially introduced into a 500 ml round-bottom flask equipped with a mechanical stirrer and a reflux condenser. The reaction mixture was stirred at 90°C for 24 h, and pure 2-vinylfuran could be directly extracted from the reaction medium. After completion of the reaction, the mixture was cooled, and the reflux condenser was replaced with another condenser maintained at 100°C, with hot 1,2-ethanediol flowing through the outer jacket. The other end of the condenser was connected to a diffuser immersed in distilled water. The reaction mixture was stirred and heated to 130-140°C, causing 2-vinylfuran to distill off and bubble out of the diffuser. It was collected at the water surface as an immiscible organic layer. After separation, the 2-vinylfuran was dried over anhydrous magnesium sulfate and characterized by 1HNMR which showed no further purification was needed. The resulting product could be stored in the dark for several months without displaying any signs of breakdown or polymerization.

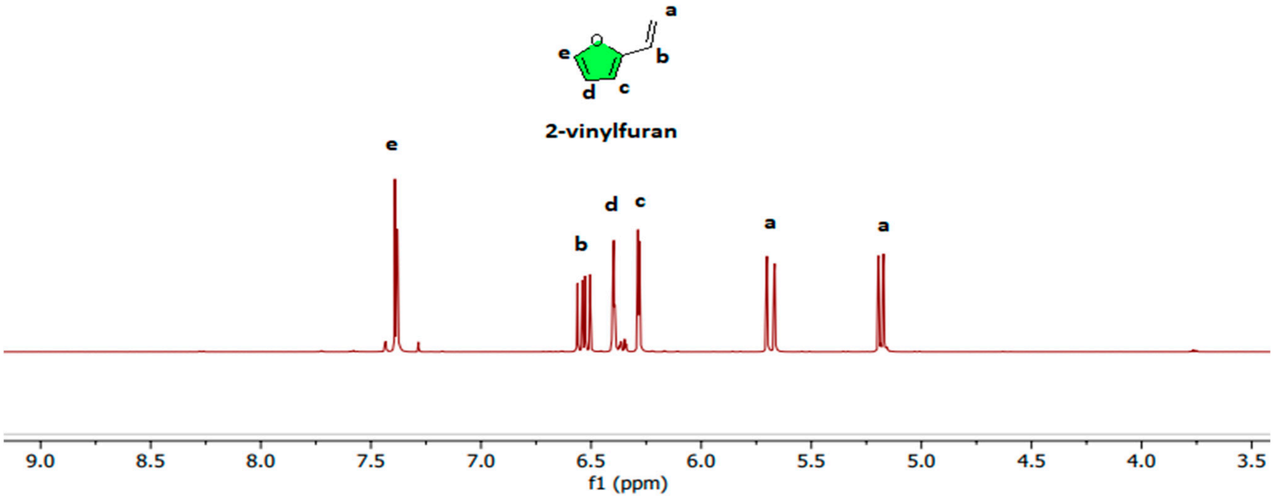

Figure S1. ^{1}H NMR of 2-vinylfuran in CDCl3

Synthesis of furan terminated polystyrene homopolymer (PS-F): The high vacuum storage tubes containing the monomer, styrene(1.5g), chlorodimethylsilane (0.1ml), 2-vinylfuran and tetrahydrofuran (THF) were attached in advance to the glass reactor. The glass reactor was connected to a high vacuum line to evacuate and refilled with

the flow of nitrogen. At first 25ml of dry purified tetrahydrofuran (THF) was transferred from high vacuum storage tube to the reactor. Then, the purified styrene(1.5gm) monomer was transferred to the reactor containing the solvent. The reactor was then cooled with a liquid nitrogen/isopropyl alcohol bath to reach -78 °C. 0.5 mL of *sec*-butyllithium (1.4 M) in cyclohexane, which was rapidly introduced into the reactor through rubber septum. A characteristic red color, indicating the formation of living polymer chains, was observed. The polymerization was allowed to continue for 1.5h at -78 °C. The reaction was terminated by transferring 0.1ml of purified dimethylchlorosilane. After 30 minutes 2ml of reaction mixture was pulled by syringe for ^{1}H NMR and FTIR characterization before adding 0.3gm 2-vinylfuran monomer to the reactor. Under continuous dry nitrogen flow to the reactor, 3-5 drops of Speier's catalyst (H_2PtCl_6) and 5 mol% of TEMPO dissolved in dry THF were added and the reaction mixture and the reaction was conducted for another 24 hours at room temperature to complete the hydrosilylation reaction. Then the resulted polymer was precipitated into a large excess of methanol. It was dried in a vacuum oven at room temperature to produce 1.6g furan functionalized polystyrene homopolymer.

Synthesis of furan functionalized Polydimethysiloxane(PDMS-F): Furan functionalized PDMS is also synthesized by high vacuum living anionic polymerization technique. The high vacuum storgae tubes containg hexamethylcyclotricyloxane(D3), chlorodimethylsilane(CDMS), 2-vinylfuran(2VF), and tetrahydrofuran were connected to the reactor in advance. The reactor was placed under a high vacuum and refilled several times with dry nitrogen. The valve of tube containing dry THF was let open to transfer 25ml of the solvent to the reactor. Then the valve of the storage tube containing D3(2gm) dissolved in THF let open to transfer 4ml of the solution to transfer in the reactor. 0.5 ml of sec-Butyllithium solution in cyclohexane was rapidly introduced in the reactor to initiate the ring opening polymerization of D3. After 15 hours, the reaction was terminated by CDMS. 2ml of solution was pulled out from the reaction mixture for ATR-IR and ^{1}HNMR characterization. After 30minutes, 0.3 g of 2-vinylfuran was added to the reaction mixture by opening the valve of the storage tube containing the dried monomer. 3-5 drops of Speier's catalyst (H_2PtCl_6) and TEMPO (0.1mol) dissolved in dry THF was added to the reaction mixture through rubber septum under dry nitrogen flow. After 24 hours the resulted polymer was harvested by washing with excess methanol and dried at 50° C under vacuum to get 2gm of furan functionalized PDMS.

Instrumentation:

^{1}H NMR spectra were recorded by Bruker AVANCE III 500 MHz spectrometer in solution states at room temperature using deuterated chloroform (CDCI3) as the solvent.

ATR-FTIR spectra were recorded by a Perkin-Elmer Spectrum 2 spectrometer to verify the functional groups of the samples and Origin pro software was used to process and analyze the data.

Perkin-Elmer's Pyris 4000 thermogravimetric instrument was used to perform thermogravimetric analysis of the samples. The decomposition temperature of all the polymers was determined at a heating rate of 10°C/min by placing the samples in ceramic pans. The analyses were performed using nitrogen flow and scanned from room temperature to 700 °C.

A thermal analysis of the samples was carried out using Perkin Elmer DSC 6000 instrument at a heating rate of 10 °C min^{-1} under a nitrogen atmosphere. In TA hermetic aluminum sample pans, all samples were collected by weighing 5-10 mg and were then hermetically sealed. The glass transition temperatures of PS and the melting temperatures of PDMS were obtained between the temperature ranges of -80 °C to 200°C. The thermal transition temperatures were reported from the second heating cycle.

UV-Vis's absorption spectra were recorded on a Perkin Elmer lambda 365 spectrophotometer.

SEM images of un-crosslinked and crosslinked samples were recorded by Zeiss Evo10 Scanning electron microscope. All the samples were sputtered with gold before imaging.

AFM height images of the samples prepared by drop-casting the solution of the samples from different solvents on a silicon wafer followed by air-drying were obtained using a Bruker Dimension Fast Scan Atomic Force Microsco

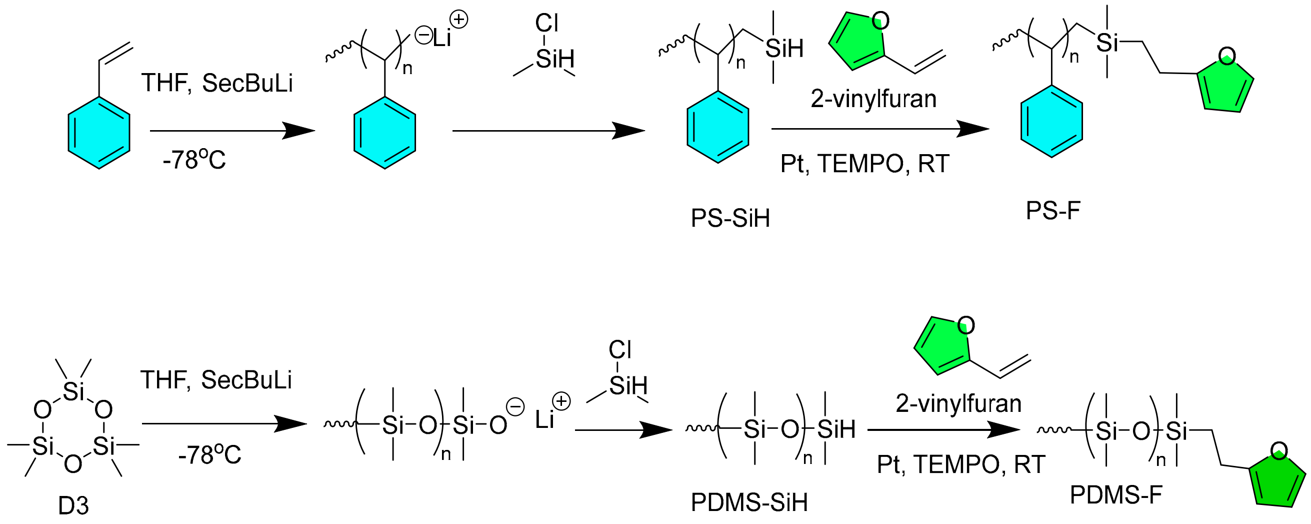

Scheme S1: One-pot synthesis of PS-F and PDMS-F

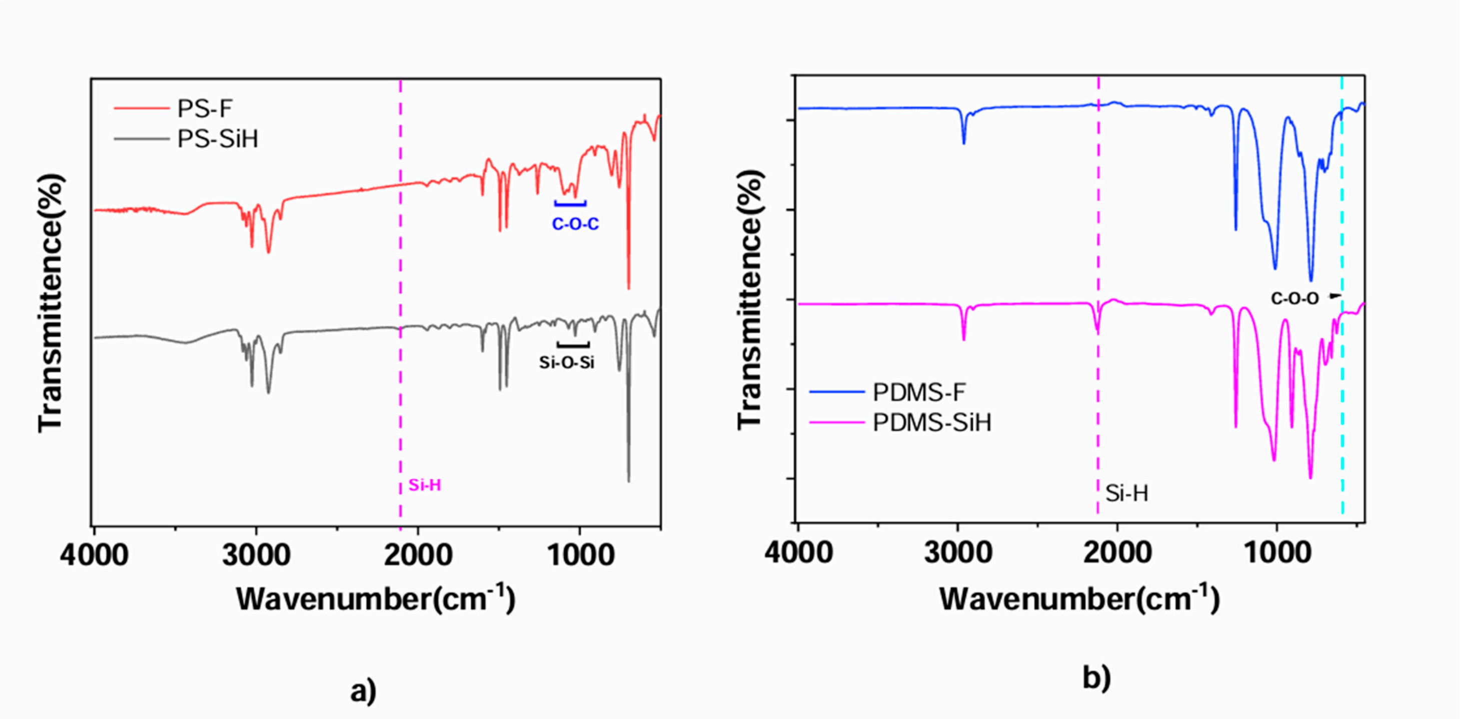

Figure S2. ATR-FTIR spectra of a) PS-F and PS-SiH, b) PDMS-F and PDMS-SiH

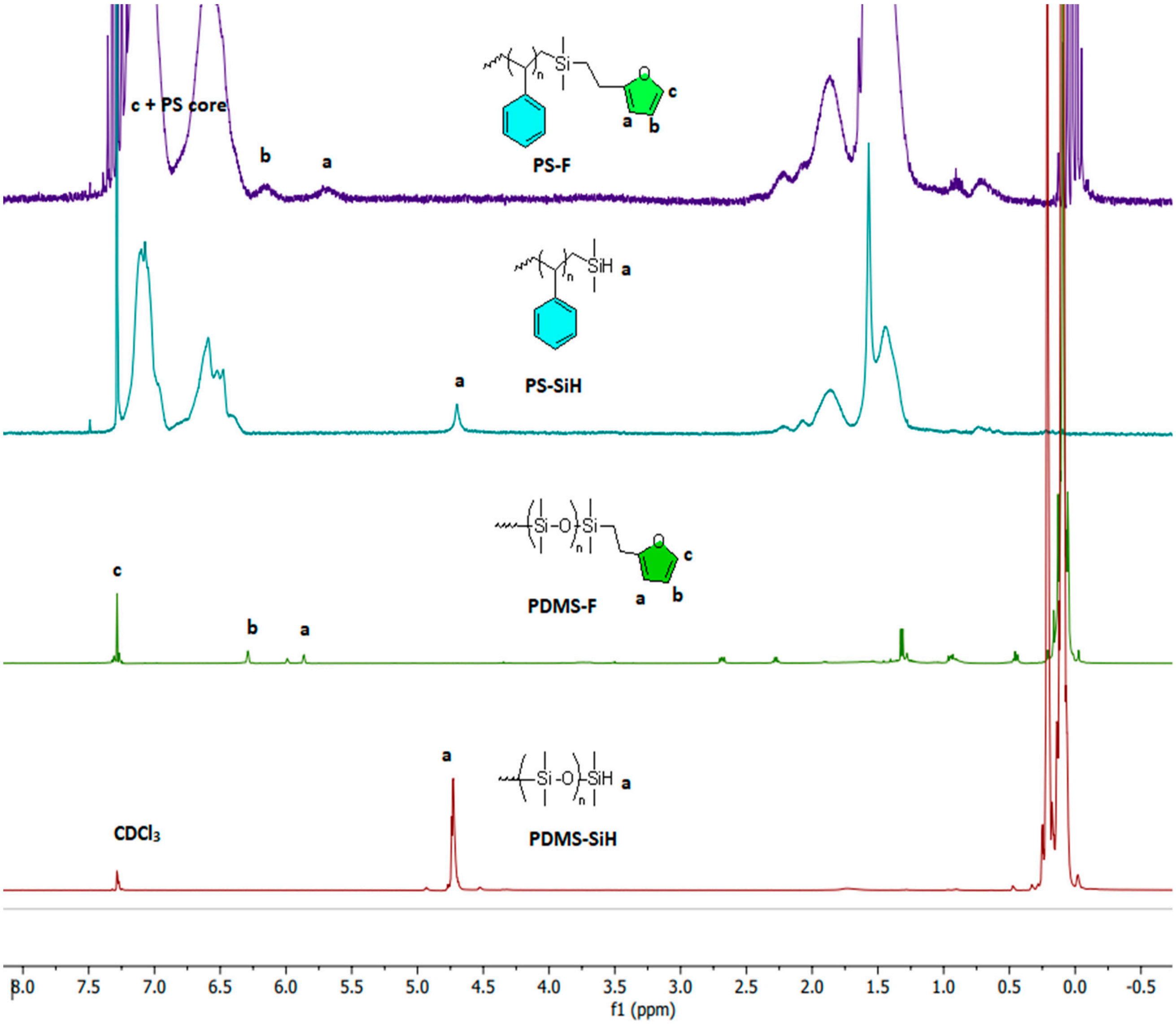

Figure S3. ^{1}H NMR of PS-F, PS-SiH, PDMS-F and PDMS-SiH

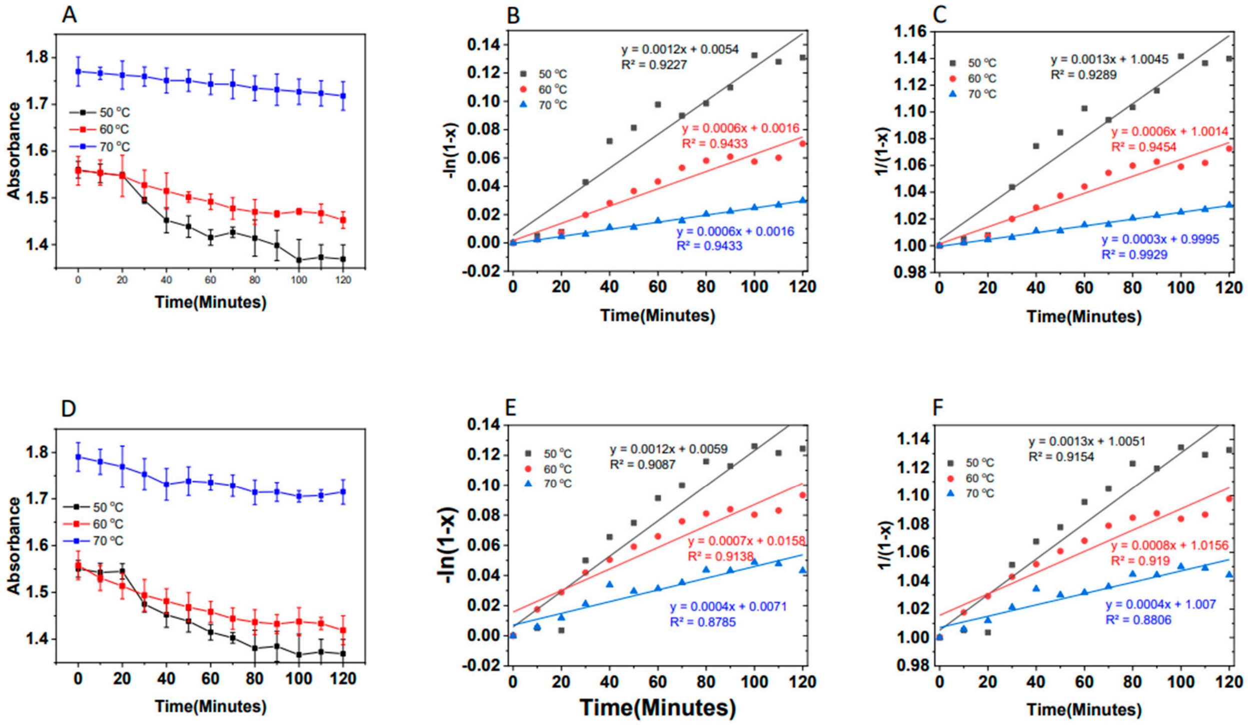

Figure S4. (A) UV-vis absorbance of PS-F;(B,C) First and second-order reaction kinetics of the PS- DA reaction studied by UV-Vis spectra; (D) UV-Vis absorbance of PDMS-F,(E,F) First and second-order reaction kinetics of the PDMS- DA reaction studied by UV-Vis spectra.

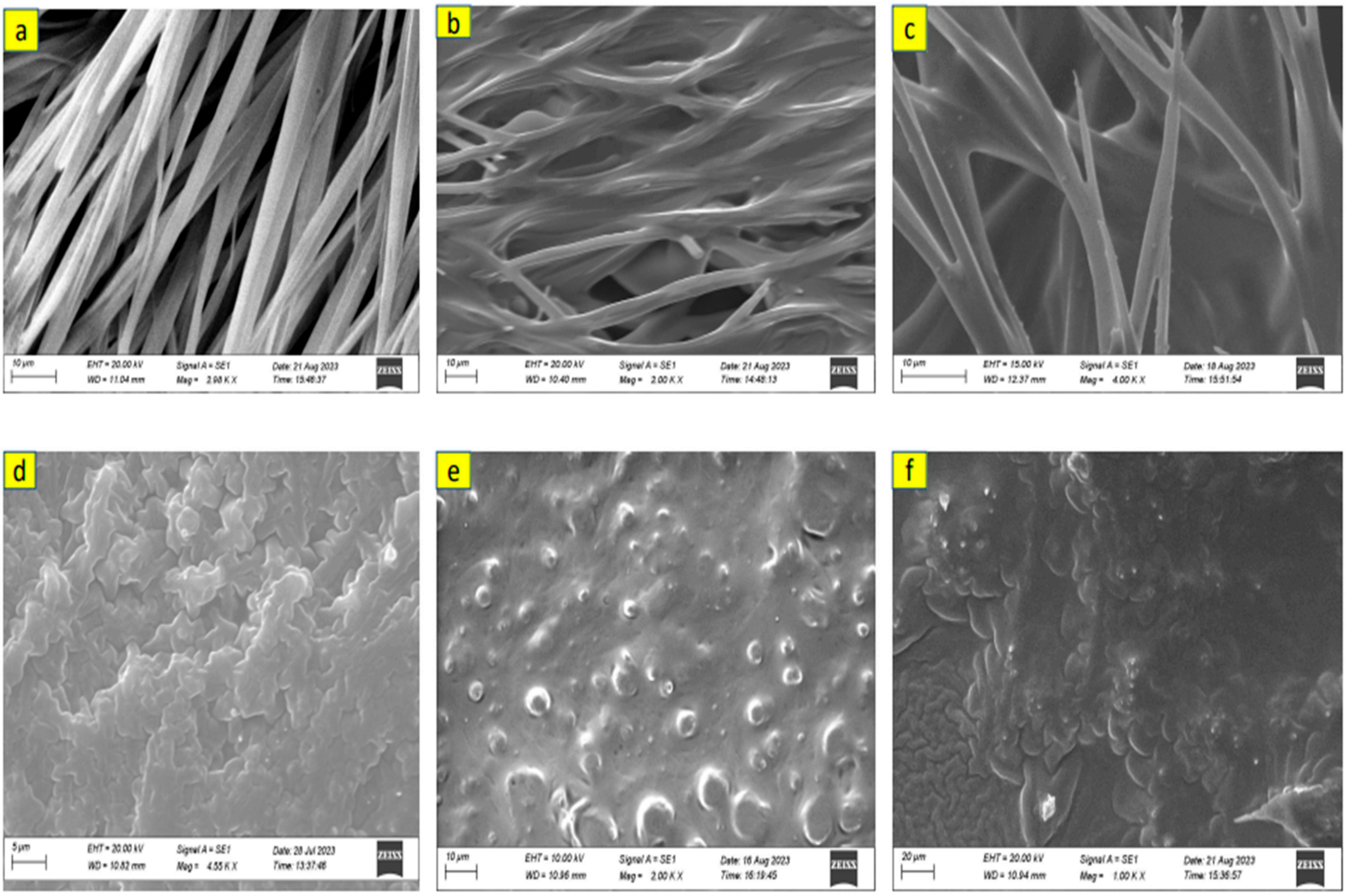

Figure S5. SEM images of (a, d) before Diels-Alder reactions, (b, e) after Diels-Alder reactions and (c, f) after retro Diels-Alder reactions of PS-F and PDMS-F with BMI.
